# Systemic Lipopolysaccharide Challenge Induces Inflammatory Changes in Rat Dorsal Root Ganglia: An Ex Vivo Study

**DOI:** 10.3390/ijms232113124

**Published:** 2022-10-28

**Authors:** Franz Nürnberger, Daniela Ott, Rebecca Claßen, Christoph Rummel, Joachim Roth, Stephan Leisengang

**Affiliations:** 1 Institute of Veterinary Physiology and Biochemistry, Justus Liebig University Giessen, Frankfurter Strasse 100, 35392 Giessen, Germany; 2 Center for Mind, Brain and Behavior (CMBB), Philipps University Marburg & Justus Liebig University Giessen, 35032 Marburg, Germany; 3 Institute of Medical Psychology and Behavioral Immunobiology, Center for Translational Neuro- and Behavioral Sciences (C-TNBS), University Hospital Essen, University of Duisburg-Essen, Hufelandstrasse 55, 45147 Essen, Germany

**Keywords:** hyperalgesia, sickness behavior, pain, LPS, cytokines, nociceptor, sensitization, peripheral nervous system, Ca^2+^ imaging

## Abstract

Inflammatory processes within the peripheral nervous system (PNS) are associated with symptoms of hyperalgesia and allodynia. Pro-inflammatory mediators, such as cytokines or prostaglandins, modulate the excitability of nociceptive neurons, called peripheral sensitization. Here, we aimed to examine if previously reported effects of in vitro stimulation with lipopolysaccharide (LPS) on primary cell cultures of dorsal root ganglia (DRG) reflect changes in a model of LPS-induced systemic inflammation in vivo. Male rats were intraperitoneally injected with LPS (100 µg/kg) or saline. Effects of systemic inflammation on expression of inflammatory mediators, neuronal Ca^2+^ responses, and activation of inflammatory transcription factors in DRG were assessed. Systemic inflammation was accompanied by an enhanced expression of pro-inflammatory cytokines and cyclooxygenase-2 in lumbar DRG. In DRG primary cultures obtained from LPS-treated rats enhanced neuronal capsaicin-responses were detectable. Moreover, we found an increased activation of inflammatory transcription factors in cultured macrophages and neurons after an in vivo LPS challenge compared to saline controls. Overall, our study emphasizes the role of inflammatory processes in the PNS that may be involved in sickness-behavior-associated hyperalgesia induced by systemic LPS treatment. Moreover, we present DRG primary cultures as tools to study inflammatory processes on a cellular level, not only in vitro but also ex vivo.

## 1. Introduction

Dorsal root ganglia (DRG) contain cell bodies of primary nociceptors that are responsible for the detection of potential environmental or internal harm [1]. Noxious stimuli (e.g., heat, cold, chemical irritants, mechanical forces) activate specific receptors of sensory neurons, such as TRP channels (transient receptor potential) [2,3]. The TRPV1 channel is activated by capsaicin, an active component of chili peppers, as well as by heat and other noxious stimuli [4]. One consequence of inflammatory stimulation of the peripheral nervous system (PNS) is an altered neuronal responsiveness upon noxious stimulation, e.g., via post-translational modulations on the TRPV1 channel involved in peripheral sensitization [5]. Such modulatory capacities have been shown for several inflammatory mediators, such as cytokines, prostaglandins, or growth factors [5]. However, mechanisms of peripheral sensitization have mainly been investigated in models of peripheral inflammatory pain, such as complete-Freund’s-adjuvant (CFA)-induced paw edema [6,7,8] and arthritis [9,10] or in different animal models of neuropathic pain [11].

Interestingly, the impact of systemic inflammation on the PNS, including DRG, has been less intensively investigated. In states of systemic inflammation, symptoms of sickness behavior occur [12]. Among lethargy, depression, social withdrawal, anorexia, and fever these symptoms also include hyperalgesia and allodynia [13,14,15]. Experimentally, sickness behavior can be induced by an injection of bacterial lipopolysaccharide (LPS) in humans [16,17,18] and animals [19,20] and, therefore, represents a well-characterized translational approach to study neuro-immune interactions [21]. However, the physiological responses to systemic LPS challenges depend on the applied dose of endotoxin [22]. While a moderate dose of 0.1 mg/kg induces rather mild signs of sickness behavior in rats, including fever [23], a high dose of 5 mg/kg results in a more severe form of sepsis and induces hypothermia [24]. In such severe cases, for example, cyclooxygenase (COX)-1 seems to play a more important role than COX-2, especially in the manifestation of hypothermia instead of fever, and thus, hypoalgesia might develop instead of hyperalgesia [22]. In both cases, regulated host responses lead to beneficial effects to fight infection. The initiation and orchestration of the sickness response is mediated by central nervous structures via three main signaling pathways of the immune-to-brain axis: humoral, neuronal, and immune-cell mediated [14,25,26]. However, symptoms of sickness behavior-associated hyperalgesia and allodynia are also evoked by modulations within the PNS, including DRG [27,28,29,30]. 

We and others have previously implemented primary cell cultures of DRG to study direct effects of LPS on the expression and release of inflammatory mediators by resident cells (neurons, satellite glial cells, macrophages), activation of inflammatory signaling cascades in distinct cell types, and neuronal responsiveness upon thermal and noxious stimulation (e.g., heat, capsaicin) [31,32,33,34,35]. However, it remains a matter of debate to which extent these direct LPS effects can be observed in states of systemic inflammation in vivo, e.g., induced by an intraperitoneal injection of LPS. 

In the present study, we aimed to characterize effects of a systemic challenge with a moderate dose of LPS (0.1 mg/kg) on the expression of inflammatory mediators in DRG, as well as the neuronal responsiveness and activation of inflammatory transcription factors after cultivation of DRG cells. Moreover, we wanted to assess if these responses reflect previous results using LPS stimulation of DRG primary cell cultures in vitro [31,32,36].

## 2. Results

**Figure 1:**
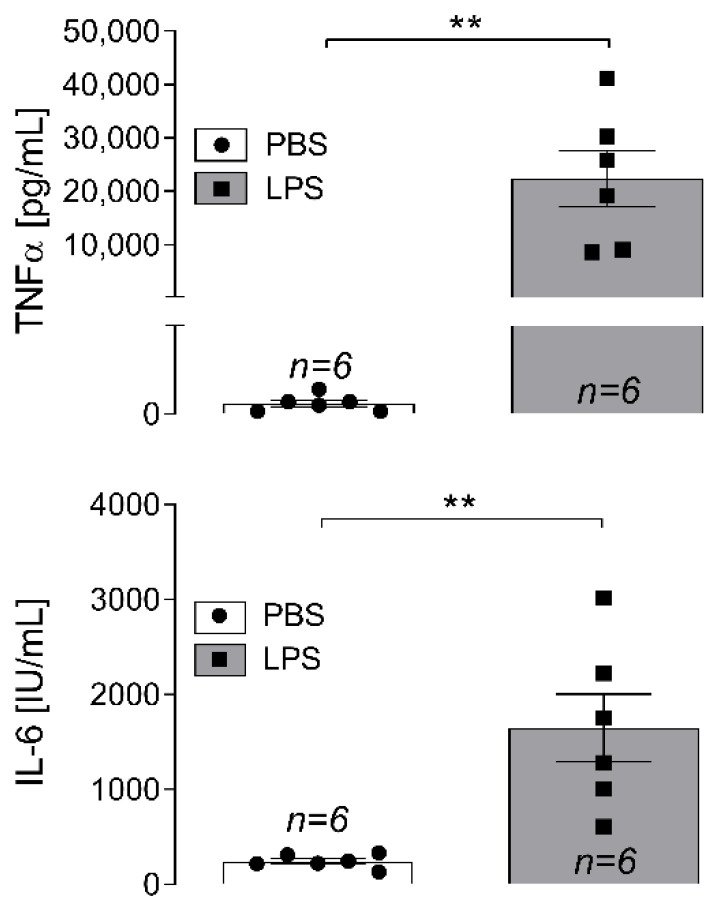
Circulating plasma cytokine levels in rats treated with PBS (controls) or LPS. Rats treated with LPS (100 µg/kg, i.p.) show higher plasma concentrations of TNFα (**A**) and IL-6 (**B**) than PBS-treated controls 3 hours after injection. Cytokine measurements were performed by means of specific bioassays. Bars represent the mean ± SEM with symbols indicating results of single animals (*n* = 6). **: *p* < 0.01. TNFα: tumor necrosis factor alpha, IL-6: interleukin-6, PBS: phosphate buffered saline, LPS: lipopolysaccharide.

**Figure 2:**
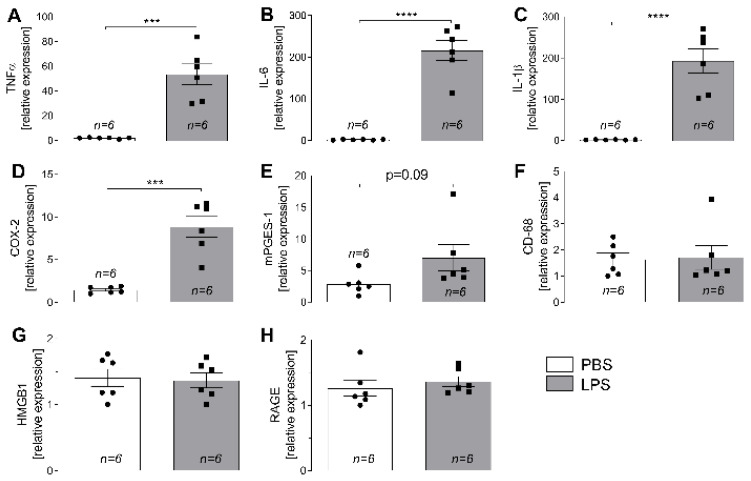
Upregulation of inflammatory target genes in lumbar dorsal root ganglia (DRG) of LPS-treated rats. RNA extracts from lumbar DRG (L4–L6) of PBS- or LPS-treated rats were pooled and used for RT-qPCR experiments to study effects on inflammatory gene expression. An elevated mRNA-expression of pro-inflammatory cytokines TNFα (**A**), IL-6 (**B**), and IL-1β (**C**) was detectable in DRG 3 hours after i.p. LPS injection. Moreover, expression of COX-2, an enzyme involved in PGE2-synthesis, was upregulated (**D**). No significant effects were detectable for mPGES-1 (**E**), CD-68 (**F**), HMGB1 (**G**), or RAGE (**H**). Bars represent the mean ± SEM with symbols indicating results of single animals (*n* = 6). ***: *p* < 0.001; ****: *p* < 0.0001. TNFα: tumor necrosis factor alpha, IL: interleukin, COX-2: cyclooxygenase-2, mPGES-1: microsomal prostaglandin E synthase-1, CD-68: cluster of differentiation 68, HMGB1: high mobility group box-1, RAGE: receptor for advanced glycation end products, PBS: phosphate buffered saline, LPS: lipopolysaccharide.

**Figure 3:**
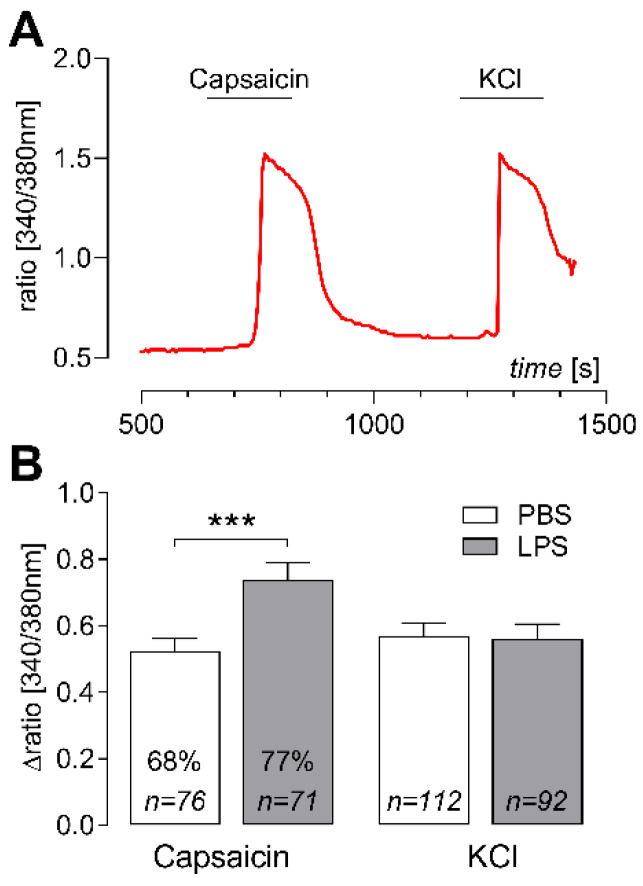
Elevated calcium responses of cultured dorsal root ganglia (DRG) neurons after LPS challenge (ex vivo). Primary cultures of DRG were obtained from PBS- and LPS-treated rats and used for Ca^2+^ imaging experiments. Capsaicin, a TRPV1 agonist and KCl were applied as excitatory stimuli leading to distinct Ca^2+^ responses (**A**). Stimulus-induced increases from baseline were calculated as Δratio [340/380 nm] (**B**). Capsaicin-responses were significantly higher in cultured DRG neurons from LPS-treated rats compared to PBS-treated controls; 68% (PBS, *n* = 76) or 77% (LPS, *n* = 71) of all investigated neurons were responsive to capsaicin-stimulation. No treatment effects were detectable for KCl-responses. Bars represent the mean ± SEM. ***: *p* < 0.001. PBS: phosphate buffered saline, LPS: lipopolysaccharide.

**Figure 4:**
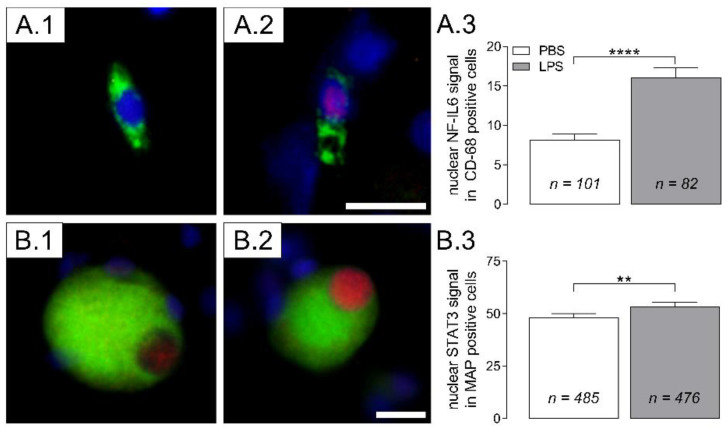
Nuclear translocation of inflammatory transcription factors nuclear factor interleukin 6 (NF-IL6) and signal transducer and activator of transcription 3 (STAT3) in cultured dorsal root ganglia (DRG) cells. Immunocytochemistry was performed in cultured DRG cells obtained from PBS- and LPS-treated rats to examine activation of inflammatory transcription factors. (**A**) In CD-68-positive macrophages (green) an enhanced nuclear signal of NF-IL6 (red) was detectable in rats treated with LPS (**A.2**) compared to controls (**A.1**). Calculating the mean nuclear intensity of the signal in the area of the nucleus (blue), a significant increase is detectable (**A.3**). (**B**) The STAT3 signal (red) was mainly detectable in MAP-positive neurons (green) of DRG cultures of LPS-treated animals (**B.2**) and controls (**B.1**). Calculating the mean nuclear intensity of the signal in the area of neuronal nuclei (blue), a significant difference is detectable (**B.3**). Bars represent the mean ± SEM. ‘*n*’ represents the number of investigated cells of the respective cell type. Scale bars present 10 µm. **: *p* < 0.01; ****: *p* < 0.0001. PBS: phosphate buffered saline, LPS: lipopolysaccharide.

LPS-induced systemic inflammation not only resulted in enhanced levels of circulating cytokines tumor necrosis factor (TNF)α and interleukin (IL)-6 in plasma samples (Figure 1) but was also accompanied by an increased expression of inflammatory marker genes in L4-L6 DRG (Figure 2) when compared to phosphate buffered saline (PBS)-treated counterpart controls. Cultured DRG neurons from rats treated with LPS showed enhanced Ca^2+^ responses upon stimulation with capsaicin than DRG neurons derived from in vivo PBS-treated animals (Figure 3). Finally, activation of inflammatory transcription factors was detectable in cultured DRG neurons (signal transducer and activator of transcription 3; STAT3) and macrophages (nuclear factor interleukin 6; NF-IL6) upon in vivo LPS stimulation (Figure 4).

### 2.1. Systemic Inflammation Induces Increase in Circulating Cytokine Concentrations

Intraperitoneal injection of LPS results in a robust systemic inflammatory response associated with symptoms of sickness behavior [19]. This is accompanied by enhanced concentrations of circulating inflammatory cytokines, such as TNFα and IL-6 [37,38]. To examine if LPS-injected animals had undergone this systemic response, plasma samples were collected from the left ventricle immediately post mortem for subsequent determination of circulating cytokine levels. Rats that had been treated with LPS showed elevated concentrations of pro-inflammatory cytokines in plasma samples (Figure 1A,B): TNFα (pg/mL): PBS: 24.00 ± 7.57 vs. LPS: 22,343.50 ± 5179.56 (mean ± SEM), **: *p*= 0.0015; IL-6 (IU/mL): PBS: 243.17 ± 29.31 vs. LPS: 1648.33 ± 357.46; **: *p* = 0.0029. 

### 2.2. LPS-Induced Changes in Expression of Inflammatory Marker Genes in L4-L6 DRG

To examine a suggested impact of systemic inflammation on the peripheral nervous system, lumbar DRG L4 to L6 were isolated unilaterally after systemic LPS challenge for 3 h and immediately shock-frozen in liquid nitrogen for subsequent examination of inflammatory gene expression. Rats injected with LPS showed an enhanced relative expression of pro-inflammatory cytokines TNFα, IL-6, and IL-1β compared to PBS controls (Figure 2A–C): TNFα: PBS: 1.99 ± 0.21 vs. LPS: 53.51 ± 8.44, ***: *p* < 0.001; IL-6: PBS: 2.14 ± 0.39 vs. LPS: 216.1 ± 23.98, ****: *p* < 0.0001; IL-1β: PBS: 1.84 ± 0.29 vs. LPS: 193.2 ± 29.70, ****: *p* < 0.0001. Moreover, we observed an upregulation of COX-2 (Figure 2D: PBS: 1.46 ± 0.15 vs. LPS: 8.85 ± 1.22, ***: *p* < 0.001), while changes in the expression of mPGES-1 remained below the level of significance (Figure 2E: PBS: 2.88 ± 0.65 vs. LPS: 7.03 ± 2.11, *p* = 0.089). Finally, no LPS effects were detectable for cluster of differentiation 68 (CD-68; PBS: 1.63 ± 0.25, LPS: 1.74 ± 0.46), high mobility group box-1 (HMGB1; PBS: 1.41 ± 0.13, LPS: 1.37 ± 0.11), or the receptor for advanced glycation end products (RAGE; PBS: 1.26 ± 0.12, LPS: 1.36 ± 0.08) (Figure 2F–H).

### 2.3. Elevated Capsaicin-Induced Ca^2+^ Responses in Cultured DRG Neurons of LPS-Treated Rats

Cultivation of DRG cells was performed to study neuronal Ca^2+^ responses upon stimulation with capsaicin, a TRPV1 agonist activating putative nociceptors, and KCl, as a vitality test for all excitable cells (neurons) (Figure 3A). A total number of 112 neurons of the PBS-treated group were investigated, of which 76 neurons responded to capsaicin (~68%). In DRG cultures from LPS-treated rats, a total population of 92 neurons was studied with 71 neurons showing capsaicin responses (~77%). The peak response from baseline of each cell was calculated as Δratio [340/380 nm], reflecting the respective elevation in intracellular Ca^2+^ concentrations ([Ca^2+^]_i_). Capsaicin-responsive neurons of LPS-treated rats showed higher elevations in [Ca^2+^]_i_ upon noxious stimulation compared to neurons from animals of the PBS control group (Figure 3B: PBS: 0.52 ± 0.04 vs. LPS: 0.74 ± 0.05, **: *p* = 0.0019). Responses to KCl did not differ between PBS- and LPS-treated groups (Figure 3B: PBS: 0.57 ± 0.04, LPS: 0.56 ± 0.05).

### 2.4. Nuclear Translocation of Inflammatory Transcription Factors (NF-IL6, STAT3) in Cultured DRG Cells

Exposition to inflammatory mediators, such as LPS, TNFα, or IL-6, induces activation of intracellular signaling cascades, including nuclear translocation of inflammatory transcription factors, such as NF-IL6 or STAT3 [39,40,41]. Therefore, we examined nuclear signal intensities of these transcription factors in distinct cellular populations, such as neurons (MAP2a + b positive cells) and macrophages (CD-68-positive cells). The transcription factor NF-IL6 was mainly detectable in CD-68-positive macrophages with an enhanced nuclear NF-IL6 signal in macrophages of LPS-treated rats (Figure 4(A.1–A.3): PBS: 8.18 ± 0.75 vs. LPS: 16.07 ± 1.26, ****: *p* < 0.0001). Signals of STAT3 were mainly co-localized with nuclei of MAP2a+b positive neurons in both treatment groups. However, nuclear intensities were enhanced in neurons of LPS-treated animals compared to PBS control groups (Figure 4(B.1–B.3): PBS: 48.26 ± 1.75 vs. LPS: 53.51 ± 1.80, **: *p* = 0.0029).

## 3. Discussion

The presented study results provide evidence for inflammatory changes and enhanced neuronal capsaicin responses in rat DRG induced by a systemic LPS challenge. Elevated plasma levels of circulating cytokines were accompanied by an enhanced expression of pro-inflammatory mediators and enzymes in lumbar DRG. Interestingly, elevated capsaicin-induced Ca^2+^ responses and activation of inflammatory transcription factors were still detectable in cultured DRG cells after 18 h of cultivation. Therefore, our study emphasizes an impact of systemic inflammation on the peripheral nociceptive system and a putative role in hyperalgesia, as one symptom of LPS-induced sickness behavior. Moreover, we emphasize DRG primary cultures as useful tools to study inflammatory processes on a cellular level ex vivo.

Systemic application of LPS results in symptoms of sickness behavior in several species including rats [37,41], mice [40,42,43], guinea pigs [41,44,45], and humans [46] and therefore represents a valuable translational model [47]. One common feature of systemic LPS challenges across species are elevated levels of circulating pro-inflammatory cytokines (e.g., TNFα, IL-6) in plasma samples, as observed in the present study. These circulating mediators represent one important pathway in the immune-to-brain communication to induce symptoms of sickness behavior, e.g., via circumventricular organs in the brain [19,25,39] or brain endothelial cells [48,49]. These symptoms include fever, anorexia, fatigue, malaise, or depression. In addition, hyperalgesia has been documented as a sign of the sickness response [17,50,51]. In the present study, we aimed to examine effects of a systemic LPS challenge on dorsal root ganglia, where cell bodies of primary nociceptors are located, responsible for the detection and transduction of nociceptive information from the periphery to the spinal dorsal horn [1]. We detected a systemic inflammation-associated upregulation of pro-inflammatory cytokines, TNFα, IL-6, and IL-1β as well as an enhanced expression of COX-2 in lumbar DRG. An elevated expression of classical pro-inflammatory mediators in DRG has regularly been observed in animal models of inflammatory pain, such as CFA-induced paw edema and is associated with neuroinflammatory processes involved in peripheral sensitization [52,53]. Via binding to the respective receptors on nociceptive neurons and activation of intracellular signaling cascades, TNFα [54], IL-6 [55,56], and IL-1β [57] modulate neuronal excitability upon noxious stimulation contributing to peripheral sensitization. Similar capacities have also been detected for PGE2 [58,59,60]. Cyclooxygenase-2 and mPGES-1 are two key enzymes in the synthesis of PGE2 and upregulated in DRG cells under inflammatory conditions in vivo [61,62] and in vitro [32,35,63]. In line with these studies, the presented data indicate a local increase in the expression of important pro-inflammatory cytokines (TNFα, IL-6, IL-1β) and enzymes (COX-2) in DRG that might directly or indirectly affect nociceptive signaling.

To study effects of LPS-induced systemic inflammation on the neuronal responsiveness of DRG nociceptors, we performed Ca^2+^ imaging experiments in cultured DRG neurons from LPS- and PBS-treated rats. Activation of the TRPV1 channel by heat or agonists, such as capsaicin, represents one important mechanism in the detection of peripheral noxious stimuli [2,3]. In the present study, we found 68% (PBS) or 77% (LPS) of capsaicin-responsive neurons, which is comparable to previous studies using DRG primary cell cultures [31,33,64]. Capsaicin responses were higher in cultured DRG neurons from rats that had previously been treated with LPS. This effect might be mediated via direct LPS-binding on toll-like receptor (TLR)-4 receptors on nociceptors [35,65,66] or indirectly via locally enhanced expression of pro-inflammatory mediators, such as IL-6, TNFα, or PGE2, which in response can sensitize nociceptors [56,59,60,67].

By means of immunocytochemistry we detected enhanced nuclear signals of the inflammatory transcription factor STAT3 in neurons, indicative of its activation. However, it has to be noted that nuclear STAT3 signals were also detectable in DRG neurons from control animals. Increased nuclear translocation of STAT3 has previously been observed in DRG neurons upon inflammatory stimulation in vitro [31,36] as well as after peripheral inflammation [68] or nerve injury in vivo [69]. Activation of STAT3 can be induced by IL-6 [70,71] and is involved in the upregulation of functional TRPV1 receptors [72]. Therefore, the enhanced levels of circulating IL-6 after in vivo LPS challenge, together with a local upregulation of IL-6 expression in LPS-stimulated DRG, could contribute to STAT3 activation in nociceptive neurons and modulate neuronal capsaicin responses. Finally, we studied activation of macrophages in DRG cultures by means of immunocytochemistry and observed a translocation of the transcription factor NF-IL6 in resident macrophages (CD-68-positive cells) 3 hours after LPS-injection. NF-IL6 is phosphorylated and, thereby, activated by mitogen-activated protein kinases in response to several inflammatory mediators, including LPS, IL-1β, or TNFα and contributes to the upregulation of genes for pro-inflammatory mediators, including IL-6 or COX-2 [73]. Therefore, an enhanced nuclear translocation in macrophages can be interpreted as an activation of this cell type [39,40]. It has previously been shown that DRG macrophages are a main source of LPS-induced pro-inflammatory mediators, such as TNFα, in vitro [31] and in vivo [54]. In the context of hyperalgesia, an enhanced activation and infiltration of CD-68-positive macrophages has been observed in animal models of arthritis-associated pain [9], peripheral nerve injury [74], and paclitaxel chemotherapy-induced peripheral neuropathy [75]. However, investigating the relative expression of CD-68, we could not detect significant effects upon LPS treatment. Therefore, we assume that the observed acute upregulation of pro-inflammatory mediators rather depends on resident than infiltrating cells.

Overall, the results of this study indicate a significant impact of inflammatory processes in DRG which may contribute to sickness-behavior-associated hyperalgesia. Showing similar results after in vivo LPS challenge compared to previous results after in vitro LPS stimulation, we present primary cell cultures of DRG as useful tools to study effects of inflammatory processes on a cellular level ex vivo.

## 4. Materials and Methods

### 4.1. Animals

All animals in the present study were 5-week (±3 days)-old male Wistar rats obtained from an in-house breeding colony weighing between 140 and 220 g and kept in groups of two rats for the experiment. A total number of 12 animals was included in this study (LPS: *n* = 6; PBS: *n* = 6). Parental animals were obtained from Charles River WIGA (Sulzfeld, Germany) and breeding, animal care, and experimental setup were performed according to the German Law on Animal Welfare, authorized by the Justus-Liebig University of Giessen and reported and approved by the regional authority of Hessia (GI 18/2 No. 69_2020). Artificial light was turned on from 07:00 a.m. to 07:00 p.m., room temperature was kept at 22 ± 1 °C, and relative humidity at 50 ± 5%.

### 4.2. Treatments and Experimental Protocol

Stock solutions of LPS (20 µg/mL; *E. coli* O111:B4, L2630, Lot: 089M4016V, Sigma-Aldrich Chemie GmbH, Taufkirchen, Germany) in sterile PBS (Sigma-Aldrich Chemie GmbH) were prepared and deep-frozen. Three days prior to injection, two animals were separated and monitored daily (weight, general condition, spontaneous behavior). Per experiment, one rat was injected intraperitoneally (i.p.) with LPS (100 µg/kg, 5 mL/kg), while the other rat was injected with the same volume of PBS, as control. Three hours after injection, the animals were sacrificed by cervical dislocation after exposure to carbon dioxide. Intracardial blood was obtained post mortem to investigate circulating levels of pro-inflammatory cytokines. The vertebral column was extracted and opened lengthwise. Dorsal root ganglia from the thoracic and lumbar segment were used for subsequent cultivation, while L4–L6 DRG were preserved in liquid nitrogen and stored at −80 °C for real-time (RT)-qPCR experiments.

### 4.3. RT-qPCR

All reagents for RT-qPCR were purchased from Applied Biosystems (Foster City, CA, USA) if not reported otherwise. From both animals (LPS and PBS) lumbar ganglia (L4–L6) were used for extraction of RNA and subsequent RT-qPCR experiments. Each DRG was homogenized separately in 200 µL RA1-buffer and 4 µL TCEP applying tissue grinders (Roth, Karlsruhe, Germany). Extraction of RNA was performed according to the NucleoSpin RNA XS Kit protocol (Macherey Nagel, Düren, Germany). After RNA purification, extracts of L4-L6 DRG of each animal were pooled to gain sufficient amounts of RNA. Later, RNA concentrations of all samples were equalized to 25 ng/µL. The following reverse transcription of RNA was performed by the use of 50 U murine leukemia virus reverse transcriptase, 10 mM deoxynucleoside triphosphate (dNTP) mix and 50 µM random hexamers in a total reaction volume of 20 µL. Relative quantification by real-time PCR was performed in triplicates of all samples on a StepOnePlus Real-Time PCR system. A TaqMan PCR Master Mix and a pre-optimized primer/probe mixture (TaqMan Gene Expression Assay) were used. For quantification the following thermo-cycling protocol was applied: polymerase activation, 50 °C for 2 min; denaturation, 95 °C for 10 min; and 40 cycles of 15 s denaturation at 95 °C followed by 1 min of annealing and elongation at 60 °C. Relative quantification was performed using the 2^−(ΔΔCt)^ method. Beta-actin (Rn00667869_m1) was used as reference gene after comparing different housekeeping gene candidates and in line with previous studies [31]. The sample values for each gene represent x-fold difference from a control sample, given a designated value of 1 within the same experiment whilst considering the housekeeping gene. The following gene expression assays were used:

TNF-α: Rn99999017_m1, IL-6: Rn01410330_m1, IL-1β: Rn00580432_m1, COX-2: Rn00568225_m1, mPGES-1: Rn00572047_m1, CD-68: Rn01495634_g1, HMGB-1: Rn0566331_m1, RAGE: Rn00584249_m1.

### 4.4. Preparation of DRG Primary Cell Cultures

Primary cultures of DRG were prepared from both rats as previously described in detail [31,32,33]. Briefly, about 20 DRG from the thoracolumbar segment were isolated, enzymatically digested, and mechanically dissociated. Cells were re-suspended and cultured in flexiPERM (micro12, Sarstedt AG & Co. KG, Nuembrecht, Germany) bounded chambers on poly-L-lysine (0.1 mg/mL, Bio&Sell GmbH, Feucht, Germany) treated glass cover slips with complete medium, consisting of Neurobasal A medium supplemented with 2% B27, penicillin (100 IU/mL)/streptomycin (0.1 mg/mL) and 2 mM L-glutamine (all from: Thermo Fisher GmbH, Darmstadt, Germany). After 4 hours of cultivation in a humidified atmosphere of 5% CO_2_ and 95% air at 37 °C medium was changed again to remove cellular debris. After cultivation overnight, DRG primary cultures were used for further experiments.

### 4.5. Cytokine Measurements (TNFα, IL-6)

Three hours after injection of LPS or PBS, the animals were sacrificed by cervical dislocation and intracardial blood was obtained to investigate circulating levels of the pro-inflammatory cytokines TNFα and IL-6. For cytokine measurements, highly sensitive bioassays were applied [76,77]. The IL-6 bioassay is based on a dose-dependent growth stimulation of IL-6 on the B9 hybridoma cell line. Serial dilutions of samples and different concentrations of an international standard (human IL-6 code 89/548, National Institute for Biological Standards and Control) were incubated for 72 h and evaluated using the dimethylthiazol-diphenyl tetrazolium bromide (MTT; Carl Roth GmbH, Karlsruhe, Germany) assay. The detection limit for this assay was 3 international units (IU) of IL-6. The TNFα bioassay is based on the cytotoxic effect of TNFα against a mouse fibrosarcoma cell line WEHI 164 subclone 13. Serial dilutions of samples and different concentrations of an international standard (murine TNFα code 88/532, National Institute for Biological Standards and Control, South Mimms, UK) were incubated for 24 h. After incubation the number of vital cells was quantified using the colorimetric MTT assay. The detection limit for the TNFα bioassay was 6 pg/mL.

### 4.6. Measurement of Intracellular Calcium ([Ca^2+^]_i_)

After 18 h of incubation in a humidified atmosphere of 5% CO_2_ and 95% air at 37 °C, DRG primary cultures were used for measurements of [Ca^2+^]_i_ as previously described [31,34]. Briefly, cells were loaded with 2 µM fura-2-AM diluted in complete Neurobasal A medium for 45 min in a humidified atmosphere of 5% CO_2_ and 95% air at 37 °C. Intracellular calcium measurements were performed under an inverted microscope (IMT-2; Olympus GmbH, Hamburg, Germany) in an individually customized Teflon^®^ culture chamber. During the experiment, cells were superfused with Ca^2+^ imaging buffer consisting of 130 mM NaCl, 5 mM HEPES, 5 mM KCl, 1.25 mM CaCl_2_, 1.0 mM MgCl_2_, and 10 mM D-glucose (all: Sigma-Aldrich Chemie GmbH), at pH 7.4 with a flow rate of 2.0 mL/min. To study stimulus-induced responses of DRG neurons the TRPV1 agonist capsaicin (1 µM) and KCl (50 mM; both: Sigma-Aldrich Chemie GmbH) were applied for 180 s. Fluorescence measurements were performed using a filterwheel-based excitation system and analyzed with MetaFluor 7.7.8.0 software (Visitron GmbH, Puchheim, Germany). The time course of emitted fluorescence (>515 nm) after alternating excitations at 340 and 380 nm was recorded by a Spot Pursuit digital CCD-camera (Model 23.0, Visitron GmbH). The 340/380 nm ratios proportional to [Ca^2+^]_i_ were computed and analyzed. An increase in the Δratio [340/380 nm] of more than 0.05 was considered a stimulus-induced Ca^2+^ response and included in evaluations.

### 4.7. Immunocytochemistry

For immunocytochemical identification of cell types, the following monoclonal antibodies or polyclonal antisera were used: CD-68 for macrophages (mouse anti rat-CD-68; 1:1000; AbD Serotec, Oxford, UK) and microtubule-associated protein 2a+b for neurons (mouse AP-20 anti-MAP2a+b; 1:600; Sigma-Aldrich Chemie GmbH). Furthermore, antibodies for the inflammatory transcription factors NF-IL6 (rabbit anti-NF-IL6, Santa Cruz, CA, USA, 1:4000) and STAT3 (rabbit anti-STAT3, Santa Cruz, 1:4000) were applied. All applied antibodies have previously been characterized in DRG primary cultures [31,32,33].

The implemented protocol for immunocytochemical examination is based on previous studies [31]. Obtained cell cultures of LPS- or PBS-treated rats were fixed with 4% paraformaldehyde (PFA) in PBS (Sigma-Aldrich Chemie GmbH) for 20 min. After three washing steps a blocking buffer, consisting of 10% fetal calf serum (FCS, Capricorn Scientific GmbH) diluted in PBS-T containing 0.05% Triton X-100 (Sigma-Aldrich Chemie GmbH) was added. Afterwards, cell cultures were incubated in primary antibodies diluted in blocking buffer for 24 h at room temperature in a humidified chamber. Incubation in secondary fluorophore-coupled antisera diluted in blocking buffer was performed for 2 h (Cy3-conjugated donkey anti-rabbit IgG (H + L), Dianova GmbH, Hamburg, Germany, 1:1000; Alexa 488 donkey anti-mouse IgG (H + L), Life Technologies GmbH, 1:500). For staining of nuclei, we used 4′,6-diamidine-2′-phenylindole dihydrochloride (DAPI; 1:10,000, Mobitec GmbH, Goettingen, Germany) for 8 min. After three final washing steps, cells were embedded in a glycerol/PBS solution (Citifluor Ltd., London, UK) on microscope slides. Cell cultures were investigated and photographed using a fluorescence microscope (BX-50, Olympus Optical, Hamburg, Germany) and the appropriate filter sets. Using the corresponding software (MetaMorph microscopic imaging software, Molecular Devices, San Jose, CA, USA) we quantified the average intensities of the inflammatory transcription factors (NF-IL6 and STAT3) within the region of interest (nuclei of the respective cell types). Brightness, contrast, and color balance were adjusted for an improved visualization of signals in the microphotographs in Figure 4.

### 4.8. Evaluation and Statistics

All results were analyzed and illustrated using Microsoft Excel 2016 (Microsoft Corporation, Redmond, WA, USA) and GraphPad Prism 9.0 software (GraphPad Software Inc., LaJolla, CA, USA). Standard distribution of all values per group was examined using the Shapiro–Wilk test. Comparison of normally distributed groups was performed with unpaired *t* tests (plasma cytokines, gene expression), while the Mann–Whitney test was applied as nonparametric test (Ca^2+^ imaging, immunocytochemistry). In all figures results are presented as mean ± SEM.

## Data Availability

The datasets generated and analyzed during the current study are available from the corresponding author on reasonable request.
